# Supplementary material for: All-at-once RNA folding with 3D motif prediction framed by evolutionary information
Source: Nat Methods. 2025 Oct 3;22(10):2094–106. doi: 10.1038/s41592-025-02833-w (PMC12510878; doi:10.1038/s41592-025-02833-w)
Supplement: Supplementary file 3 — Source code to run CaCoFold-R3D. Data and results. [file 41592_2025_2833_MOESM3_ESM.gz › supplemental_material/results/CaCoFold-R3D/CaCoFold-R3D_Rfam/RF01984_ZEB2_AS1_1.R2R.sto.pdf]

RF01984_ZEB2_AS1_1

5′-C U A U A A A A G A U G U A A C A G A U G C A A A C U G U A A ● A C A A G G G C ● A U U A A C C C U U U C U C U G C C ● R G C A ● R C U ● ● R U ○ C C Y A C ● C Y C Y R C A C C U A U U G U C U Y C Y ● U G C Y R G R A G C U U U G C R G A A A A C C U G G A A
